# Controversial issues on rare subcutanous hydatid disease

**DOI:** 10.1016/j.ijscr.2019.03.011

**Published:** 2019-03-20

**Authors:** Rosario Vecchio, Salvatore Marchese, Eva Intagliata

**Affiliations:** Department of General Surgery and Medico- Surgical Specialties, University of Catania, Italy

Dear Editor,

We read with great interest the case report published by Salih et al. [1] on International Journal of Surgery Case Reports 2018; 51: 8–10. It is a unique case of concomitant multiple hydatid cysts of thigh and pelvis. This case, however, might raise debate on some aspects, which we would like to share with the Authors and the readers of your journal.

In the reported case, liver and lungs were not affected. Since it is well recognized that humans can accidentally become intermediate hosts ingesting eggs, what would be the way of infestation in this case? Could parasites skip the portal-liver tough filter by using lymphatic or venous shunts and reach directly the systemic circulation? Alternatively, could we hypothesize a direct subcutaneous contamination through an injured skin? In the case described by Battyani et al. [2], a wasp sting has been correlated to a cyst formation. In a study that we recently published [3], a patient with a solitary subcutaneous hydatid cyst of the deltoid region had a shoulder trauma in his history compatible with a direct contamination. Notwithstanding, he did not mention any skin injury related to his trauma. Was the case reported by Salih et al. [1] queried about an alleged direct infestation of the thigh?

In case of subcutaneous hydatid disease, it is still unclear whether blood tests have a role in the diagnosis. In our review [3], parasitological serology like ELISA test or other specific antibodies immunodiagnostic tests confirmed the diagnosis only in few cases. Considering the fact that a negative result will never exclude the diagnosis, should we still consider them in the diagnostic pathway? Or should we follow Salih et al. and choose not to use them?

Surgical excision can be associated with spillage of hydatid contents in the surgical field [4]. To the best of our knowledge, differently than for visceral disease, it has never been reported that a subcutaneous cyst has triggered any septic or allergic reaction [3]. In the case reported by Salih et al., however, it needs to be pointed out that the cyst was detected in the pelvis. According to our experience, on the treatment of intra-abdominal hydatid disease, we believe that preventive sterilization of the hydatid cyst should be mandatory. This can be accomplished by a neo-adiuvant antiparasitic therapy. Alternatively or in association, we aspirate intraoperatively the cystic fluid with a needle and subsequently we push hypertonic saline solution. We always prefer to wait few minutes after this step before completing the excision [5].

## Conflicts of interest

There is no conflict of interest.

## Funding

There are no sources of funding for this research.

## Ethical approval

This is a letter to the editor which doesn’t require any ethical approval.

## Consent

There is not a study on patients or volunteers. Therefore it doesn’t require any consent.

## Author contribution

All the Authors have contribuited to the design, data collection, data analysis, interpretation, writing the paper.

## Registration of research studies

There is a letter to the editor, which does’t imply new human studies, and no registration.

## Guarantor

Eva Intagliata, MD PhD University of Catania, Italy.

## Provenance and peer review

Not commissioned, externally peer-reviewed
